# A view on the skin–bone axis: unraveling similarities and potential of crosstalk

**DOI:** 10.3389/fmed.2024.1360483

**Published:** 2024-03-04

**Authors:** Tadatsugu Morimoto, Hirohito Hirata, Kazunari Sugita, Permsak Paholpak, Takaomi Kobayashi, Tatsuya Tanaka, Kinshi Kato, Masatsugu Tsukamoto, Shun Umeki, Yu Toda, Masaaki Mawatari

**Affiliations:** ^1^Department of Orthopaedic Surgery, Faculty of Medicine, Saga University, Saga, Japan; ^2^Division of Dermatology, Department of Internal Medicine, Faculty of Medicine, Saga University, Saga, Japan; ^3^Department of Orthopedics, Faculty of Medicine, Khon Kaen University, Khon Kaen, Thailand; ^4^Department of Neurosurgery, International University of Health and Welfare Narita Hospital, Chiba, Japan; ^5^Department of Orthopaedic Surgery, Fukushima Medical University, Fukushima, Japan

**Keywords:** skin-bone axis, crosstalk, bone metabolic disease, osteoporosis, diffuse idiopathic skeletal hyperostosis

## Abstract

The phrase “skin as a mirror of internal medicine,” which means that the skin reflects many of the diseases of the internal organs, is a well-known notion. Despite the phenotypic differences between the soft skin and hard bone, the skin and bone are highly associated. Skin and bone consist of fibroblasts and osteoblasts, respectively, which secrete collagen and are involved in synthesis, while Langerhans cells and osteoclasts control turnover. Moreover, the quality and quantity of collagen in the skin and bone may be modified by aging, inflammation, estrogen, diabetes, and glucocorticoids. Skin and bone collagen are pathologically modified by aging, drugs, and metabolic diseases, such as diabetes. The structural similarities between the skin and bone and the crosstalk controlling their mutual pathological effects have led to the advocacy of the skin–bone axis. Thus, the skin may mirror the health of the bones and conversely, the condition of the skin may be reflected in the bones. From the perspective of the skin–bone axis, the similarities between skin and bone anatomy, function, and pathology, as well as the crosstalk between the two, are discussed in this review. A thorough elucidation of the pathways governing the skin–bone axis crosstalk would enhance our understanding of disease pathophysiology, facilitating the development of new diagnostics and therapies for skin collagen-induced bone disease and of new osteoporosis diagnostics and therapies that enhance skin collagen to increase bone quality and density.

## 1 Introduction

The phrase “skin as a mirror of internal medicine,” has long been known (1). In other words, the skin is an indicator of the body’s response to various diseases and can serve as an offshoot or surrogate biomarker for diagnosis, indicating symptoms of visceral diseases such as metabolic, gastrointestinal, and neoplastic diseases (2). In some cases such as palmoplantar pustulosis, psoriasis, and reactive arthritis (Reiter’s syndrome); skin lesions and osteoarthritis may occur together, indicating a close association between skin diseases and bone and joint pathologies via an inflammatory mechanism. Because skin findings may reflect bone joint disease, the phrase “skin as a mirror of bone joint disease” may be warranted.

The occurrence of degenerative bone metabolic diseases, including osteoporosis, and proliferative bone diseases, such as diffuse idiopathic skeletal hyperostosis (DISH) and posterior longitudinal ligament ossification (OPLL), have increased in recent years owing to the rapid aging of the population (3–5).

To reduce the socioeconomic burden as well as the burden on patients and healthcare providers, elucidating the pathogenesis of the disease for early diagnosis and treatment is indispensable. Osteoporosis results in bone loss, whereas DISH and OPLL are osteoproliferative diseases characterized by a specific pattern of ossification of the spinal ligaments. Despite seemingly conflicting pathologies, both are associated with metabolic syndrome and are influenced by the systemic endocrine and immune systems and low-level inflammation (3–5). In addition, sex, genetics, aging, metabolism, inflammation, lifestyle, and gut microbiota affect bone metabolism and skin conditions (5, 6).

The skin acts as a barrier between the environment and the internal environment and plays an important role in maintaining homeostasis. To maintain skin homeostasis and recover the structural and functional integrity of lesioned skin, skin cells produce hormones and neurotransmitters (7). Through neurotransmitters, the skin is included in a signaling axis between the brain and intestine that regulates their respective functions. In addition, the skin is the only organ that produces vitamin D (VD). VD is also involved in immune system physiology, regulation of other hormonal activities, protection against numerous types of neoplasms, maintenance of skeletal muscles, carbohydrate metabolism, cardiovascular and reproductive systems, neurocognition, bone metabolism, and skin metabolism. Therefore, the skin is considered an endocrine organ (7).

Conventionally, the bone is thought to be an inert organ that stores calcium and phosphate to serve the body. In reality, the bone is a dynamic organ, with coordinated bone formation exerted by osteoblasts and resorption by osteoclasts. Osteoblasts are involved in regulating the systemic body under the influence of several local and systemic factors. Thus, recently, bone has been redefined not only as a skeleton that sustains the body and protects internal organs but also as an active endocrine organ. Consequently, the bone has been demonstrated to engage in crosstalk with other organs (i.e., muscles, brain, gut, immune system, blood vessels, pancreas, kidneys, liver, and gonads) and is involved in systemic homeostasis to regulate organ activity (8). Thus, a crosstalk between the bone and many other organs has been identified; however, there are few reports evaluating the crosstalk between the bone and skin.

In addition, similar mechanisms between fibroblasts and keratinocytes in the skin and osteoblasts in the bone can produce hyaluronic acid and periostin, which exert multiple functions, including tissue repair, anti-inflammatory activity, anti-aging activity, and immunomodulation (9, 10).

Due to the anatomical similarities between skin and bone, in which collagen is a major component, there have been reports of an association between skin thickness and bone mineral density (BMD) (11). Skin and bone also have functional similarities in terms of endocrine functions and provide protection from external stimuli (6, 12). Additionally, several reports have claimed that skin-derived materials affect bone metabolism and bone-derived materials affect systemic metabolism (13, 14).

Despite the contrasting phenotypes of the soft skin and hard bone, they share many similarities (6), further indicating the possibility of systemic crosstalk (13). Based on the similarities and crosstalk between skin and bone, we propose the comprehensive concept of the “skin–bone axis,” referring to skin–bone crosstalk, which would help understand the relationship between the skin and bone and to elucidate pathological conditions. In addition to the biological interest, the skin–bone axis might help provide valid suggestions for better skin and bone conditions or provide unexpected answers to questions of anatomy and immunology. From the perspective of the skin–bone axis, the similarities between skin and bone anatomy, function, and pathology, as well as the possibility of crosstalk between the two, will be unraveled in this review.

To expand the scope of the review, allowing access to additional studies, and improve flexibility, we utilized a narrative review approach and analyzed several key papers on skin–bone correlations published in peer-reviewed scientific journals.

This review begins by summarizing studies that have investigated skin–bone correlations, outlining the common anatomical physiology and function of the skin and bone, common pathologies, and describing the evidence for possible crosstalk between skin and bone.

## 2 Correlations between skin thickness and bone mineral density

In 1965, McConkey et al. (15). described older women with osteoporotic fractures often had thin skin. In 1972, Black et al. (16). documented a correlation between thin skin and osteoporosis. Since then, the association between skin thinning and osteoporosis has been investigated, and many parameters of skin–bone correlation have been used, including skin thickness, elasticity, collagen for skin parameters, and BMD for bone parameters.

Aurégan et al. (11). summarized 14 studies on skin and bone parameters in postmenopausal patients with osteoporosis in a systematic review. The years of publication were one study in the 1960s, one in the 1970s, one in the 1980s, six in the 1990s, four in the 2000s, and one after 2010. Eight studies compared skin thickness to BMD, seven of which showed significant correlations (R ranging from 0.19 to 0.486); two studies showed significant correlations between BMD and skin elasticity (R ranging from 0.44 to 0.57), and BMD and skin collagen (*R* = 0.587). Although some correlation has been shown with skin and bone parameters in postmenopausal osteoporosis, the correlation coefficients were moderate or low at best due to the problems of inconsistent skin thickness measurement sites, low accuracy of BMD measurements before 1990, and the multifactorial nature of osteoporosis and skin thinning (11). Collagen, a major component of the skin, is produced in the earliest stages of skin turnover (11, 17). Skin thickness is significantly influenced by the amount of collagen present (11, 17). However, in bone, collagen cross-linking is followed by mineralization, which leads to bone formation; therefore, the effect of factors other than collagen on bone density may be significant (11). This may explain the moderate correlation between skin thickness and bone density. Furthermore, with regard to skin thickness, it should be noted that epidemiologic studies have reported that skin tends to be significantly thicker in men than in women and that people with Asian ethnic backgrounds tend to have thicker skin than people with European ethnic backgrounds, indicating that there are sex-related and ethnicity-related differences.

Most reports describing a skin–bone association have investigated the association between skin thickness and BMD, that is, dermatoporosis and osteoporosis. Few reports have demonstrated a relationship between skin thickness and osteophytes or osteoproliferative disorders (OPLL, DISH). Imamura et al. reported that the skin of patients with ligament ossification was thick and pathological findings showed proliferation of extracellular materials binding to type I collagen fibers in the dermis layer (18).

**Figure 1:**
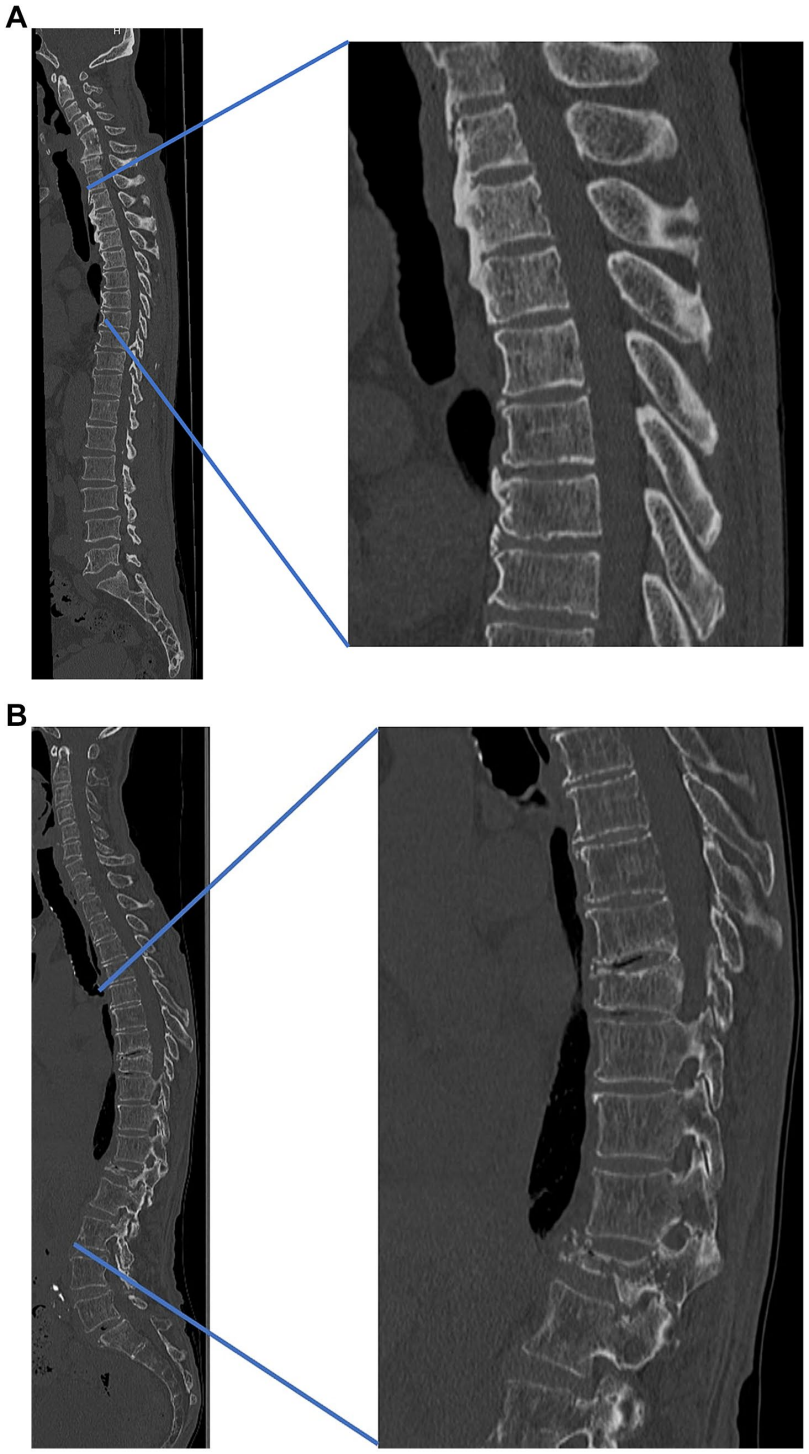
Representative cases **(A)** patient with thick skin and high bone density and **(B)** patient with thin skin and low bone density. **(A)** A 55-year-old male patient with thick skin and high bone density, cervical ossification of the posterior longitudinal ligament, and thoracic ossification of ligament flavum. Lumbar bone mineral density, 0.78 g/cm^2^. Skin thickness: cervical, 6.0 mm; thoracic, 5.8 mm; lumbar, 5.3 mm. **(B)** A 75-year-old male patient with thin skin, low bone density, and osteoporotic vertebral fracture (thoracic 9th, lumbar 1st). Lumbar bone mineral density, 0.61 g/cm^2^. Skin thickness: cervical, 2.1 mm; thoracic, 1.8 mm; lumbar, 1.9 mm. Both cases **(A,B)** are both from our institution.

Compared to osteoporotic cases, in OPLL cases, many spine surgeons may experience thickening of the skin on the back during spinal surgery (Figures 1A,B). The relationship between skin thickness and BMD, as well as osteophyte proliferation, requires further investigation.

Ultrasonography can determine skin thickness, collagen maturity, and cross-linking abnormalities (19). Ultrasound skin parameters were found to be linked to bone collagen maturation (19). The benefits of ultrasonography as a diagnostic tool are the lack of radiation exposure, fast and convenient measurements, and low cost compared with DXA or CT. With further advances in instrumentation and knowledge, skin ultrasound may become a biomarker for bone metabolic diseases, complementing the results of BMD measurements, blood analysis, and bone metabolic markers.

## 3 Similarities between skin and bone

The skin and bone have many similarities in terms of anatomy, physiology, function, and relationship with immunity (Table 1).

**Table 1:** Similarities between skin and bone.

	Skin	Bone
Anatomy	Major organic component: Collagen (type1)	Major organic component: Collagen (type 1)
Cells involved in formation Origin	Keratinocyte (epidermis), fibroblast (dermis) Mesenchymal stem cell	Osteoblast Mesenchymal stem cell
Collagen deterioration factors	Aging, corticosteroid treatment, decline of estrogen and growth hormone, and advanced glycation end products (AGEs) deposition	Aging, corticosteroid treatment, decline of estrogen and growth hormone, and advanced glycation end products (AGEs) deposition
Function	Turnover, protective, endocrine, association with the immune system	Turnover, protective, endocrine, associated with the immune system
Cells involved turnover-regulation Origin Turnover-regulating cytokines	Langerhans cell Hematopoietic stem cell WNT, RANKL, and BMP	Osteoclast Hematopoietic stem cell WNT, RANKL, and BMP
Secreted common materials	Collagen, elastin, periostin, and hyaluronic acid	Collagen, periostin, and hyaluronic acid
Potential common etiology		
Hypertrophic diseases of the skin and bone	Acromegaly and pachydermoperiostosis	Acromegaly and pachydermoperiostosis
Atrophic diseases of the skin and bone	Osteoporosis, hypopituitarism, Cushing’s syndrome, and osteogenesis imperfecta	Osteoporosis, hypopituitarism, Cushing’s syndrome, and osteogenesis imperfecta

Receptor activator of nuclear factor-kappa B ligand (RANKL). BMP, bone morphogenetic protein.

### 3.1 Anatomy, physiology, and immunology

Cells associated with skin and bone formation include keratinocytes and dermal fibroblasts (FB) in the skin, and osteoblasts (OB) in the bone (6, 12, 20). OB are fibroblasts that secrete specific materials for collagen calcification (6). Besides BMD as an indicator of bone quantity, qualitative factors, such as the structural and material properties of bone, contribute to bone strength, and collagen properties constitute an essential factor in fracture risk (19). Both keratinocytes, FB and OB, are derived from mesenchymal stem cells and produce not only collagen (primarily type I collagen), which is the main component of the skin and bone but also various materials that act locally and systemically (6, 12). Therefore, the skin and bones may functionally influence each other (6, 12).

Because both the skin and bone are collagen-based tissues, there may be a strong biochemical link between them (6, 21). Furthermore, collagen may have a common pathogenesis because it is similarly altered by common etiologies (11). In fact, collagen in the skin and bone decreases with aging, corticosteroid treatment (22), the decline of estrogen (6–8, 11) and growth hormones (12, 13), and advanced glycation end-product deposition, causing degradation of collagen cross-linking structures (23–26). Aging-induced collagen loss causes yellowing, browning, poor elasticity, deeper wrinkles, wrinkling, and thinning, reflecting the atrophy of the collagenous dermis in the skin and osteoporosis in the bones (23, 24, 27). Age-related alterations in the dermis demonstrate disruptions in the elastic fiber network and a reduction in the number of collagen fiber bundles (28). The effects of skin aging undermine the skin at a functional level as well, diminishing the important preventive properties of the skin. The effects of aging are called dermatoporosis in the skin and osteoporosis in the bones; moreover, attention has focused on the prevention and treatment of dermatoporosis (29). A causal relationship between osteoporosis and bone collagen has been suggested because collagen, as well as mineralization, is important for bone structure and strength, and the collagen content and cross-linking status in the bone have been investigated (26, 30).

Furthermore, an additional similarity is that the skin and bones constantly turn to maintain homeostasis. Langerhans cells in the skin and osteoclasts in the bone play the main roles in turnover, both of which are macrophages originating from hematopoietic stem cells (6, 31). From a genetic and functional perspective, Langerhans cells and osteoclasts have likely evolved from a common ancestor (31). Moreover, there are similarities in the cytokines that regulate skin and bone turnover: WNT, receptor activator of nuclear factor-kappa B ligand (RANKL), and bone morphogenetic proteins (BMP) (6, 31). Such similarities are also plausible because skin and bone originate from a common extraembryonic layer and growth are closely interdependent during development (6, 31). However, whether there is a relationship between the skin and bone turnover cycles and whether osteoporosis drugs that control bone turnover affect the skin have not been investigated; thus, these issues require further study.

The skin contains a variety of immune cells that continually monitor and defend organs from attack and maintain homeostasis. Skin wound healing involves a rapid and robust immune response and subsequent suppression of inflammatory signaling. Thus, the skin communicates closely with the immune system. In bone turnover, the factors involved in inflammation have been shown to be associated with important factors (6, 31).

Crosstalk between the skeletal and immune systems has long been studied. In 1972, Horton first reported the interaction between immune cells and osteocytes and discovered that immune cells stimulated by bacterial antigens produce osteoclast-activating factors (32). Immune cells play an integral role in postmenopausal osteoporosis (8). The bone and the immune system share a wide variety of molecules, including cytokines, chemokines, transcription factors, and signaling molecules (33). Furthermore, bone cells and immune cells exist in the same microenvironment in the bone marrow where hematopoietic stem cells and immune progenitor cells ultimately migrate during mammalian development (34). Thus, the term “osteoimmunology” has been proposed to describe the involvement of immune system cytokines in bone metabolism (33).

In summary, both the skin and bone are closely related to the immune system (8). They share similar constituent cells (FB-OB, Langerhans cell-osteoclast) and major components (collagen), and collagen is pathologically modified by common etiologies, such as aging and drugs. Given their close connection to the immune system through the sharing of many regulatory molecules, such as cytokines and signaling molecules, it is not surprising that crosstalk may occur after skin and bone differentiation and the overlapping processes involved in skin and bone lesions.

### 3.2 Potential common etiology

Since skin and bone share common components, primarily collagen, they may share a common pathology due to a similar pathological modification of collagen by a common etiology. Connective tissue degradation in the skin and bones cannot be isolated. Aging, drugs (i.e., glucocorticoids, androgens, estrogens, and bisphosphonates), and common genetic predispositions may produce thickening and atrophic lesions in the skin and bone (32).

Skin and bone thinning is an important clinical issue in patients receiving treatment with glucocorticoids. Glucocorticoids have negative effects on all skin compartments, including severe hypoplasia, loss of elasticity with increased tearing and fragility, telangiectasia, bruising, skin transparency, and skin barrier dysfunction (33). Glucocorticoids may cause dermal and epidermal atrophy, flattening of the dermal-epidermal junction, decreased fibroblast proliferation, and impaired collagen content in the dermis (33). Glucocorticoids dramatically reduce the rate of bone formation, osteoblast count, osteocyte count, and activity, and consequently prolong osteoclast lifespan (34). The incidence of osteoporosis has been estimated to be 30–50% in glucocorticoid-treated patients (34). In addition, acromegaly (35) and pachydermoperiostosis (36–39), which produce skin thickening (increase in skin collagen), bone hypertrophy (increase in bone density), hypopituitarism, Cushing’s syndrome, anorexia nervosa (40), and osteogenesis imperfecta, result in skin thinning and bone atrophy (35, 41, 42). Skin collagen increase/decrease correlates with BMD (27).

Pachydermoperiostosis is a rare osteoarticular and cutaneous syndrome, which can be familial or idiopathic. This disease includes thickening of the facial skin and scalp (pachyderma); hypertrophy of the extremities due to periarticular, periosteal, or subperiosteal bone formation; joint deformities; and, in some cases, neurological deficits. The widening of the transverse diameter of the bone results from increased osteogenesis, and histology has simultaneously demonstrated elevated collagen formation and elevated urinary excretion of hydroxyproline in pachydermoperiostosis (39). The responsible genes, hydroxyprostaglandin dehydrogenase (HPGD) and solute carrier organic anion transporter family member 2A1 (SLCO2A1), have been identified, leading to an understanding of the etiology and the development of treatment for skin and bone hypertrophy (37, 38). Genetics have been found to link the increase/decrease in skin collagen with the increase/decrease in bone formation.

## 4 Crosstalk between the skin and bone

**Figure 2:**
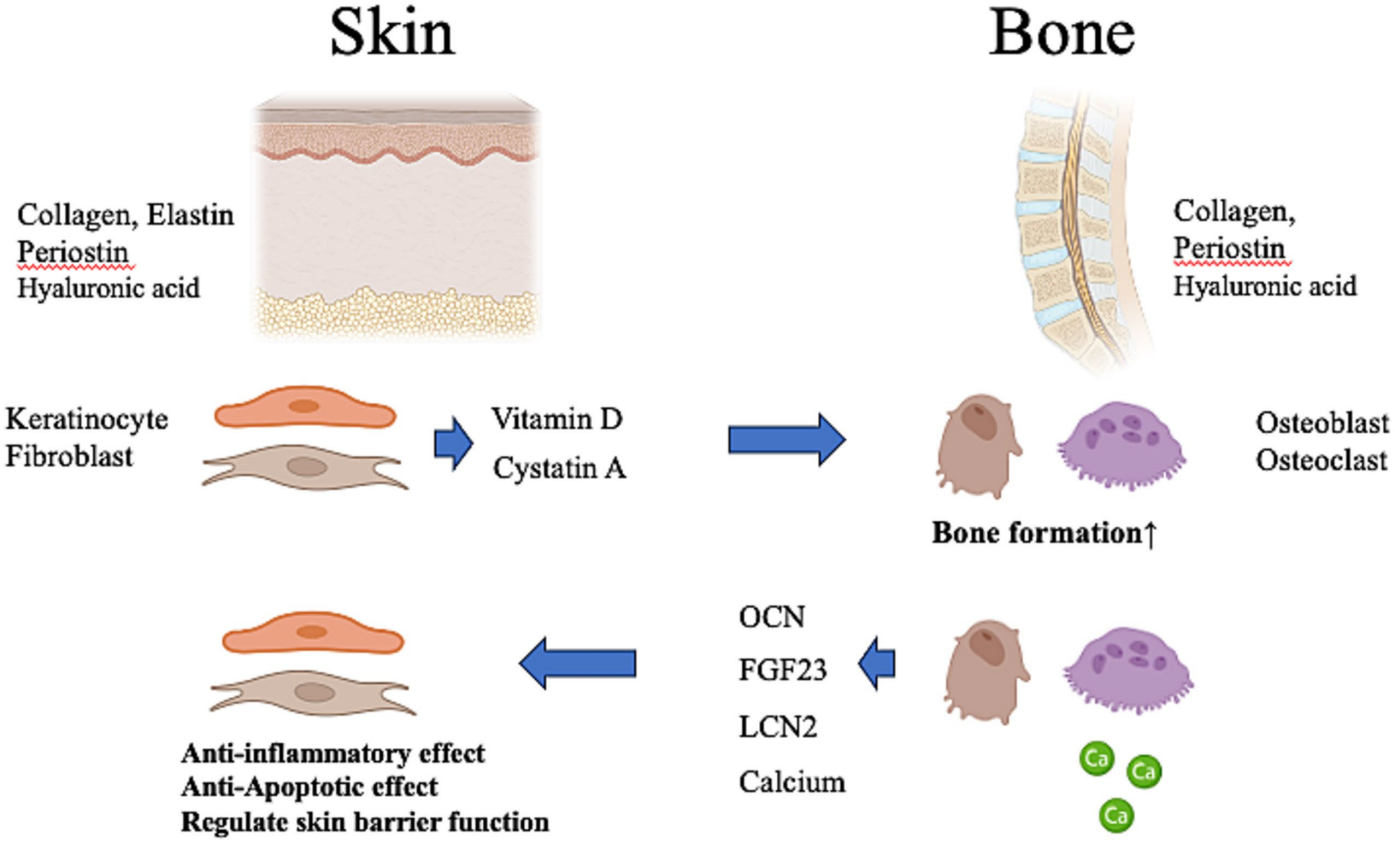
Possibility of crosstalk between the skin and bone. The skin produces vitamin D and cystatin A, which affect bone metabolism. Vitamin D deficiency of skin origin may induce systemic calcium deficiency, resulting in osteoporosis in the bone. The bone produces a variety of osteokines and calcium that influence the metabolism of the body. These contribute to anti-inflammatory and anti-apoptotic effects in the skin and regulate skin barrier function. OCN, osteocalcin; FGF23, fibroblast growth factor-23; LCN-2, lipocalin-2. This figure was created using BioRender.com.

Thus far, we have discussed the common mechanisms between skin and bone involving collagen; we will now discuss the possible crosstalk between the skin and bone (Figure 2), which can be mutually influenced by the materials produced by the skin and bone. In recent years, skin and bones have been recognized as more than just organs, producing substances that affect the entire body (43–45).

Despite the opposite phenotypes of soft skin and hard bone, several materials produced by the skin and bone can be considered hormones because of their systemic effects on the entire body via the bloodstream (6, 43–45). Thus, bidirectional endocrine and metabolic signals between the skin and bone may be necessary to ensure skin health and bone health, and conversely, skin disease may adversely affect the bone and vice versa. We will discuss below materials that may influence the bone and skin either locally or systematically.

### 4.1 From the skin to the bone

Materials secreted by skin keratinocytes, including cytokines, growth factors, antimicrobial peptides, and VD, have been implicated in a broad spectrum of biological processes that support skin function, including wound repair, inflammatory processes, and cell differentiation (46).

Oral fibroblasts exert a paracrine effect on osteoblast progenitors and restore osteogenic differentiation of mesenchymal stem cells in the presence of zoledronic acid (47).

Upon sun exposure, VD was formed in the skin via 7-dehydrocholesterol (7-DHC) by skin keratinocytes. The skin is the only organ capable of producing VD and its metabolites and is a principal target of VD. VD also affects bone metabolism (48, 49), and VD deficiency can cause rickets, osteomalacia, and osteoporosis (49). In addition, from a vertebrate evolutionary perspective, bone derives from mineralization surrounding the skin’s basement membrane, and its evolution depended on sunlight exposure and photosynthesis of VD in the skin (31). In the skin, VD contributes to collagen production by regulating keratinocyte proliferation and differentiation, the epidermal barrier function, inflammation, wound healing, and sun protection, suggesting that VD deficiency leads to dermatoporosis (29). VD has also been shown to act directly on organs such as the bone and skin or indirectly on inflammatory processes that affect the immune system and, in turn, are major factors in the onset of numerous diseases, including bone and skin aging (29).

Liang et al. (13). observed that age-related bone thinning and epidermal thinning occur simultaneously and found that early skin aging and skin atrophy promote bone loss in mice. Cystatin A is a material secreted by keratinocytes in the skin that binds to the activated C kinase 1 receptor on osteoblasts and osteoclast precursor cells, thereby promoting their proliferation and inhibiting osteoclast differentiation. Cystatin A secretion decreases with skin aging in both mice and humans, resulting in a decrease in the number of osteoblasts and osteoclasts, and causing senile osteoporosis.

### 4.2 Impact of skin disorders on bone health

Chronic inflammatory skin diseases are closely associated with bone health, with a primary focus on osteoporosis. Conditions such as psoriasis, atopic dermatitis, chronic urticaria, and bullous diseases (e.g., pemphigus vulgaris and bullous pemphigoid) are recognized as chronic cutaneous inflammatory diseases associated with osteoporosis (50) (Table 2).

**Table 2:** Summary of reported cases of association between skin and bone diseases.

References	Skin disease	Bone disease	Study design	Cases	Main results	Possible causes
Wi et al. (52)	Psoriasis	Osteopenia, osteoporosis, pathological fractures	Literature review	13 studies	Association with osteoporosis: yes 10, no 3 Inconsistent (because of small sample sizes and missing patient information): Patients with extensive psoriasis with a longer duration of psoriasis are at increased risk of osteopenia and osteoporosis.	Systematic corticosteroids, low vitamin D levels, less physically active
Ogdie et al. (53)	Psoriasis and PsA	Pathological fractures (vertebrae, hip)	Longitudinal cohort study	Psoriasis (*n* = 158,323), PsA (*n* = 9,788) aged 18–89	PsA: all fracture aHR 1.26 (1.06–1.27), mild psoriasis: all fractures, vertebral and hip fracture: aHR 1.07 (1.05–1.10), 1.17 (1.03–1.33) and 1.13 (1.04–1.22), severe psoriasis; all fracture and vertebral fracture: aHR 1.26 (1.15–1.39) and 2.23 (1.54–3.22).	Increased prevalence of risk factors for osteoporosis and fracture (e.g., diabetes, alcohol abuse, smoking, depression, antidepressant use, corticosteroids, methotrexate, and ciclosporin)
Shalom et al. (51)	Chronic urticaria	Osteoporosis	A longitudinal, community-based cohort study	Chronic urticaria (*n* = 11,944)	The adjusted multivariate analysis demonstrated that chronic urticaria was significantly associated with a higher risk for osteoporosis (HR 1.23, 95% confidence interval 1.10–1.37, *p* < 0·001).	Increased mast cell numbers are associated with increased bone resorption and decreased bone formation. Female sex, systemic corticosteroids, chronic inflammation
Silverberg (54),	Atopic Dermatitis	Low BMD	Cross-sectional study	3,049 children and adolescents aged 8–19 years old	Lower BMD z-score for the total femur (survey linear regression; adjusted β [95% CI]: −0.42 [0.68 to −0.16]), including trochanter (−0.29 [−0.54 to −0.05]) and femoral neck (−0.29 [−0.53 to −0.05]) and total lumbar spine (−0.31 [−0.52 to −0.11]).	Higher levels of IgE, WBC counts levels, and higher odds of 25-OH vitamin D deficiency, low calcium and alkaline phosphatase, dietary restrictions
Wu et al. (55)	Atopic dermatitis	Low BMD, osteopenia, osteoporosis, related fractures	Systematic review and meta-analysis	10 studies, children and adolescents and adults, Study participants ranged from 29 to 61,065,660	Adults: atopic dermatitis (OR [95% CI], *p*-value) fracture (1.13; [1.05–1.22]; *p* = 0.001) Atopic dermatitis; osteoporosis (1.95; [1.18–3.23]; *p* = 0.009), osteopenia (1.90; [1.51–2.38]; *p* < 0.001)	(1) Inflammation-induced bone loss, (2) low vitamin D levels, (3) Corticosteroids, (4) Dietary restrictions, (5) Less physical activity, (6) Depression, stress, anxiety, and sleep disturbance, (7) Obesity, cardiovascular disease, and high alcohol and tobacco consumption
Chovatiya and Silverberg (56)	Bullous disease (pemphigus and pemphigoid)	Osteopenia, osteoporosis, osteomalacia, pathological fractures	Cross-sectional study	Pemphigus (*n* = 4,506) pemphigoid (*n* = 8,864)	Pemphigus;(adjusted OR [95% CI]) Osteopenia (2.20 [1.59–3.05]), osteoporosis (2.54 [2.16–2.98]), osteomalacia (29.70 [4.05–217.83]), fractures (2.04 [1.42–2.91]) Pemphigoid; (adjusted OR [95% CI]) Osteopenia (1.59 [1.06–2.41]), osteoporosis (1.38 [1.18–1.63]), fractures (1.26 [1.04–1.53])	Systematic and topical corticosteroids, inflammation-induced bone loss, less physical activity, low vitamin D levels
Hsu et al. (57)	Bullous disease (pemphigus)	Osteopenia, osteoporosis	Case–control study	Pemphigoid (*n* = 130), age/sex-matched controls (*n* = 390)	Pemphigoid; (adjusted OR [95%CI]) osteopenia;10.07 [3.72–27.25], osteoporosis;4.19 [1.50–11.73]	Systematic corticosteroid

Ref, reference; PsA, psoriatic arthritis; PTH, parathyroid hormone; OP, osteoporosis; BMD, bone mineral density; aHR, adjusted Hazard Ratio; OR, Odds ratio; CI, confidence intervals.

Conversely, palmoplantar pustulosis (PPP) in chronic inflammatory skin diseases has been characterized by hyperostosis, which potentially indicates a specific association (58).

Factors contributing to osteoporosis in chronic skin inflammatory diseases include (1) systemic inflammation, (2) low levels of VD, and (3) therapeutic agents (50).

The well-established link between inflammation and bone loss is rooted in the understanding that under inflammatory conditions, T cells, IL-1, and tumor necrosis factor (TNF) serve as co-stimulators of osteoclastogenesis. They promote the expression of NF-κB and other transcription factors involved in bone resorption, thereby enhancing osteoclastic bone resorption (59). Both chronic inflammatory skin diseases and osteoporosis share a common inflammatory pathway characterized by elevated inflammatory cytokines such as TNF-α, INF-γ, and IL-6 (60). With regard to inflammatory cytokines, psoriasis is associated with elevated levels of TNFα, IL-23, and IL-17, with these cytokines also having a significant impact on bone erosion in psoriatic arthritis (60–62). In addition, highly effective antibody therapies (biologics) targeting these cytokines have been developed, with a growing list of new inhibitory antibodies being investigated (60, 61). Inflammatory cytokines such as TNF-α, IL-6, and IL-17 are also implicated in chronic urticaria (53), atopic dermatitis (63), and bullous diseases (64, 65). Lower levels of VD have also been reported in patients with psoriasis (66, 67), urticaria, atopic dermatitis (68), and bullous diseases (50).

Therapeutic agents used against chronic inflammatory skin diseases include systemic corticosteroids (psoriasis, urticaria, atopic dermatitis, bullous diseases), immunosuppressive agents (psoriasis), and biologics (psoriasis, PPP) (50, 68, 69). Both corticosteroids and immunosuppressive agents are associated with severe and rapid trabecular bone loss (50, 68).

As our understanding of the pathophysiology of psoriasis and PPP has advanced, biologics have been developed to inhibit the actions of inflammatory cytokines. TNFα inhibitors, IL-17 inhibitors, and IL-23 inhibitors have been introduced to treat psoriasis (70), and IL23 inhibitors (IL-1 and IL-36 inhibitors in clinical trials) to treat PPP (58, 71). The anti-inflammatory effects of biologics, such as TNF-α inhibitors (72) and IL-17 inhibitors (73), have demonstrated their efficacy in inhibiting bone resorptive destruction.

It is crucial for both dermatologists and orthopedic surgeons to be cognizant of the impact of skin diseases and their treatments on bone metabolism. With the aging population, the consequences of osteoporosis on patient morbidity, mortality, quality of life, and even the impact of socioeconomic factors are expected to increase.

### 4.3 From the bone to the skin

Osteoblasts secrete a variety of materials, including “osteokines” (14), which are bone-derived factors besides collagen (14). Osteokines, including osteocalcin (OCN), fibroblast growth factor-23 (FGF23), and lipocalin-2 (LCN-2), are important components of the endocrine system that works in close collaboration with other organs to maintain homeostatic balance and health (74).

Based on a combination of mouse genetic engineering and clinical observations, including disease symptoms and drug side effects, OCN has been identified as a novel osteokine of bone origin that regulates biological processes in multiple organs, including bone, fat, liver, muscle, pancreas, testis, and brain (75–77). OCN is a molecule of osteoblast origin coded for by the bone γ-carboxyglutamate protein gene (78). OCN levels increase during physical activity and decrease with age (8). During progressive bone resorption, the affinity of OCN for the bone materials is reduced, and OCN is released into circulation, where it acts for other organs (79). Undercarboxylated OCN may be closely associated with insulin sensitivity and glucose tolerance (14).

FGF23 is a growth factor found in bone tissue, secreted by osteoblasts and osteocytes, and regulates systemic phosphate and VD levels (80). FGF23 inhibits phosphate reabsorption in the renal tubules by reducing the production and secretion of parathyroid hormone (80). The key difference between OCN and FGF23 may be that FGF23 regulates phosphate metabolism, a process closely related to bone health, whereas OCN has many other functions (75–77).

Similar to OCN, another osteoblast-derived mediator, LCN2, is a glycoprotein that mediates insulin secretion and regulates energy metabolism by improving glucose tolerance and insulin sensitivity (8).

The potential utility of osteokines as biomarkers for cardiovascular disease has been reported, but their effects on the skin are unclear. Since glucose tolerance and the anti-inflammatory and anti-apoptotic effects of osteokines are also important for the skin, the effects of osteokines on the skin may be a subject of future investigation. Relevant combinations of skin diseases and osteokines include FGF23 and psoriasis (81), cutaneous-skeletal hypophosphatemia syndrome (82), OCN and psoriasis (83), and LCN-2 and psoriasis (84).

Furthermore, calcium (Ca) plays an important role in maintaining homeostasis of the skin barrier function (85). VD deficiency of skin origin may induce systemic Ca deficiency, resulting in osteoporosis in the bones and impaired skin barrier function, hypersensitivity, and pruritus in the skin (29, 86).

These results may help elucidate the pathogenesis of inflammatory skin diseases, as well as the mechanism of a new metabolic signaling axis between the skin and bone, and identify new therapeutic targets for the treatment of skin and bone diseases.

### 4.4 Impact of bone disease on skin health and disease

As this article has mainly focused on bone metabolism, the effects of bone metabolic diseases (osteoporosis) on skin health (disease) should also be discussed. Although no reports on the negative effects of osteoporosis on the skin have been found, several reports of anti-osteoporotic agents causing cutaneous adverse reactions have been described (50, 87–90).

Cutaneous adverse reactions associated with the use of bisphosphonate, raloxifene, parathyroid hormone and its derivatives (87), and denosumab (89, 90) have ranged from benign reactions of drug eruptions, urticaria, and cellulitis to severe cutaneous adverse reactions such as drug rashes with eosinophilia and systemic symptoms (DRESS), Stevens–Johnson syndrome (SJS), and toxic epidermal necrolysis (TEN) (50, 87). Although life-threatening cases of SJS and TEN have occurred in bisphosphonate (alendronate, risedronate, ibandronate, zoledronic acid)- and denosumab-treated patients, these are very rare (50, 87, 90). Raloxifene, a selective estrogen receptor modulator, is associated with rash, flushing, and sweating, whereas parathyroid hormone has been associated with reactions at the injection site, erythema, rash, and sweating (87). Denosumab, a human monoclonal antibody, may cause dermatitis, eczema, pruritus, and, less commonly, cellulitis (90). In general, the diagnosis of cutaneous adverse reactions is more difficult when a patient is taking multiple comorbidities and medications. Both dermatologists and orthopedic surgeons should be aware of cutaneous adverse reactions to anti-osteoporotic agents.

### 4.5 Common materials with multiple functions secreted from skin and bone: periostin and hyaluronic acid

Periostin, a common extracellular materials protein secreted by fibroblasts and osteoblasts, is involved in tissue formation, repair, fibrosis, and inflammation (91). Aberrant periostin expression has been implicated in atopic dermatitis of the skin (91), osteoporosis, and ossification of the posterior longitudinal ligament of the bone (92). Periostin induces epithelial cell proliferation and differentiation, leading to re-epithelialization (93). Periostin expression in the skin decreases with age and affects collagen production (93). In bones, periostin is preferentially expressed on osteocytes and periosteal osteoblasts, regulates the recruitment and adhesion of osteoblasts from the bone marrow and blood, and induces local bone formation in response to mechanical stress and inflammation (94). It also plays an important role in regulating bone microstructure, strength, and mass by promoting osteoblast differentiation and survival and regulating collagen fiber formation and extracellular materials assembly (94, 95).

Hyaluronic acid (HA) is a natural glycosaminoglycan. As a major component of the extracellular materials, HA plays an essential role in skin repair, cosmetics, cartilage protection, anti-inflammation, wound healing, tissue regeneration, immunomodulation, anticancer and antiproliferative effects, antidiabetes, anti-aging, and maintenance of homeostasis in the body (9, 10). HA products involving gels and creams have been reported to be remarkably effective in the treatment of a wide range of inflammatory skin diseases, and intra-articular injections have been reported to significantly reduce joint pain, synovitis, and swelling (9, 10). Since bone and skin tissues in the body are in close proximity, both periostin and HA produced in the skin or bone tissues may affect bone or skin metabolism by acting on osteoblasts or skin fibroblasts in a paracrine or endocrine manner. Periostin from skin fibroblasts has been shown to act on keratinocytes in a paracrine or autocrine manner *in vivo* and may affect osteoblasts in a paracrine or autocrine manner (96). However, no such periostin-mediated interactions between the skin and bone tissues have been reported. It is also possible that such interactions between bone and skin tissues affect bone metabolism and lead to the onset or development of related diseases.

## 5 Gut microbiota and skin and bone

There may be a skin–bone correlation mediated by a third entity, the gut microbiota. The gut microbiota (GM), a population complex of intestinal bacteria, has been implicated in metabolic homeostasis, development and maturation of the immune system, resistance to infection, and production of neurotransmitters (97, 98). Recent studies have underlined the key regulatory functions of the GM in neuroendocrine and immune functions through the activity of the microbiome and its metabolic products. GM has been involved in disease processes in various organs in and out of the gut (heart, brain, kidney, liver, skin, and bone) (97). GM dysbiosis has been linked to inflammatory diseases inside and outside the gut; including inflammatory bowel disease, cancer, rheumatoid arthritis, multiple sclerosis, diabetes, food allergies, eczema, asthma, chronic pain, obesity, and metabolic syndrome, psoriasis and atopic dermatitis in the skin, and osteoporosis and osteoarthritis in bones (17, 97–100). The relation of GM dysbiosis to skin and bone disease has been shown to be mediated by the dysfunction of the intestinal barrier, increased levels of inflammatory mediators, and metabolites released from the gut microbiota (17, 97–100). Close bidirectional correlations and potential mechanisms between gut microbiota and skin and bone health/disease have been demonstrated and proposed as the gut-skin axis (99, 101) and gut-bone axis (102–104), respectively. The skin and gut share similarities in that they have active and complex immune and neuroendocrine organs, frequent external exposure to the environment, and contain a variety of microbiomes. In addition to acting through the gut-derived immune system, indigenous skin bacteria may be influenced by bioactive compounds, such as neurotransmitters, hormones, and SCFAs (end products of gut microbial metabolism). In addition, it has also been suggested that skin inflammation can arise from minute changes in a single bacterial species of the gut microbiota (105). Bone and skin health may be compromised by the common etiologies of GM. The association between inflammatory skin and the gut microbiota has been shown to be mediated by dysfunction of the gut barrier, by elevated inflammatory mediators, and by metabolites emitted from the GM (99). The causes of GM dysbiosis include genetic predisposition, aging, drugs, diet, alcohol consumption, and smoking (17, 97–100). These factors also have a negative impact on skin and bone health. Thus, there may be a common etiology or pathology of the skin and bone, starting with the GM.

## 6 Conclusion and perspectives

As the population ages and the incidence of bone metabolic diseases continues to increase, a detailed understanding of skin–bone interactions may be the key to addressing unmet clinical needs. Despite the phenotypic differences between soft skin and hard bone, they are strongly connected to the immune system, in addition to their protective and endocrine functions. The skin and bone consist of dynamic organs in which fibroblasts and osteoblasts secrete collagen and involved in biosynthesis, while Langerhans cells and osteoclasts control the turnover. Whether there is a correlation between skin and bone turnover or whether osteoporosis drugs affect the skin as well as the bone, remains unknown and needs to be addressed in the future.

In addition, the quality and quantity of collagen in the skin and bone can be modified by aging, inflammation, estrogen, diabetes, and glucocorticoids. Skin and bone collagen are pathologically modified by aging and metabolic diseases such as drugs and diabetes. In addition to the structural similarities between skin and bone, they have endocrine functions, and materials have been reported to indicate the possibility of reciprocal crosstalk between skin and bone, leading to the proposal of a skin–bone axis.

Thus, the skin may mirror the health of the bones and, conversely, the condition of the skin may be reflected in the bones. Based on the skin–bone axis, a thorough elucidation of the pathways governing this crosstalk could lead to a better understanding of the disease pathophysiology, and skin findings could serve as biomarkers for bone metabolic diseases. This will also facilitate the development of new diagnostics and therapeutics for skin collagen-induced bone disease, as well as new osteoporosis diagnostics and therapeutics that enhance bone quality and density by enhancing skin collagen.

## Author contributions

TM: Conceptualization, Investigation, Writing – original draft, Writing – review & editing. HH: Data curation, Methodology, Visualization, Writing – original draft. KS: Project administration, Supervision, Writing – review & editing. PP: Supervision, Formal analysis, Writing – original draft. TK: Writing – original draft, Investigation. TT: Formal analysis, Investigation, Methodology, Writing – original draft. KK: Methodology, Project administration, Supervision, Writing – review & editing. MT: Writing – original draft, Conceptualization, Validation. SU: Project administration, Resources, Visualization, Writing – original draft. YT: Writing – review & editing, Conceptualization, Investigation. MM: Project administration, Supervision, Validation, Writing – review & editing.
